# Promiscuous recombination of *LoxP* alleles during gametogenesis in cornea *Cre* driver mice

**Published:** 2008-03-20

**Authors:** Daniel Y. Weng, Yujin Zhang, Yasuhito Hayashi, Chia-Yi Kuan, Chia-Yang Liu, George Babcock, Wei-Lan Weng, Sandy Schwemberger, Winston W.-Y. Kao

**Affiliations:** 1 Departments of Ophthalmology, University of Cincinnati, Cincinnati, OH; 2 Division of Developmental Biology, Cincinnati Children’s Hospital medical Center, Cincinnati, OH; 3 Department of Surgery, University of Cincinnati, Cincinnati, OH; 4 Department of Research, Shriners Hospital for Children, Cincinnati, OH; 5 Department of Cell and Cancer Biology, University of Cincinnati, Cincinnati, OH

## Abstract

**Purpose:**

To examine whether promiscuous *Cre/LoxP* recombination happens during gametogenesis in double transgenic mice carrying *LoxP* modified alleles and *Cre* transgene driven by tissue-specific promoter outside the gonads of adult mice.

**Methods:**

Cre driver mice were crossbred with reporter mouse lines (e.g., *ZEG* and *Rosa26R*) to obtain *Cre/ZEG* and *Cre/Rosa26R* double transgenic mice. The frequency of promiscuous *LoxP/Cre* recombination was determined by the expression of second reporter genes in the offspring of double transgenic mice.

**Results:**

The frequency of promiscuous *LoxP/Cre* recombination varied in different lines of Cre driver mice and in the sex of the same driver mice with higher penetrance in male than in female double transgenic mice. Polymerase chain reaction (PCR) and recombination analysis demonstrate that the recombination of floxed allele occurs during the transition from spermatogonia (diploid) to primary spermatocyte (tetraploid) in the testis. Thereby, target-floxed allele(s) may be ubiquitously ablated in experimental animals intended for tissue-specific gene deletion.

**Conclusions:**

Gametogenesis-associated recombination should always be examined in tissue-specific gene ablation studies.

## Introduction

The functions of growth factors and transcription factors in mice can be determined by analyzing the phenotypic effects in the knockout mouse models via gene targeting. However, this strategy has frequently been impeded due to systemic defects and/or embryonic lethality. The *Cre/LoxP* approach has been used to overcome these limitations in a variety of developmental systems [1]. In the tissue where Cre is expressed, the gene of interest flanked by LoxP elements is excised. This system allows the inactivation of the target gene in a tissue-specific manner and reduces the probability of embryonic lethality of the experimental animals.

In an attempt to achieve cornea-specific gene ablation using the *Cre/LoxP* system, we recently generated cornea stromal keratocyte-specific keratocan-Cre (*Kera-Cre, KC*) transgenic mice and corneal epithelium-specific keratin 12-Cre (*K12-Cre*) mice via knock-in strategy of gene targeting techniques [2]. A double transgenic *Kera-Cre/Rosa26R* mouse showed *LacZ* expression in the corneal stroma whereas a *K12-Cre/Rosa26R* mouse showed *LacZ* expression in the corneal epithelium. Both mice were the offspring derived from the mating of reporter *Rosa26R* mice with *Kera-Cre* or *K12-Cre* mice [3]. Thus, these two mouse lines *Kera-Cre* and *K12-Cre* may serve as cornea-specific Cre animal models for deleting the gene of interest in the corneal stroma and epithelium, respectively.

To build up colony size of double transgenic *Cre/Rosa26R* mice, we sibling bred *Cre/Rosa26R* double transgenic mice. Surprisingly, some of the offspring showed systemic *LacZ* expression patterns from crosses between *Cre/Rosa26R* mice; furthermore, similar unexpected *LacZ* expression was also observed in breeding between *Cre/Rosa26R* and wild type mice, suggesting the excision of *LoxP*-modified genomic DNA by promiscuous Cre recombinase activities during development. In the present study, we have examined how often and when such promiscuous Cre activation may take place in Cre driver mouse lines targeted for the use of tissue-specific gene ablation. *Wnt1-Cre*, *Kera-Cre*, *K12-Cre*, and *BF1-Cre* driver mice were chosen because they are used in our ongoing research projects. Our data suggested that Cre recombinase was activated during gametogenesis and led to germ line ablation of a *LoxP*-modified allele in zygotes in *Kera-Cre*, *K12-Cre*, and *BF1-Cre* mice but not *Wnt1-Cre* mice.

**Figure 1:**
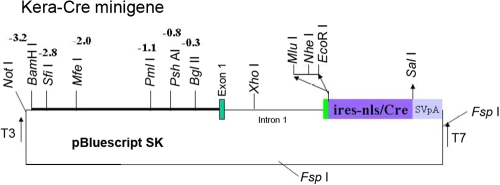
Diagram of *KC* (*Kera-Cre*) minigene construct. An expression DNA construct was prepared with pBskII plasmid (Strategen, La Jolla, CA) using conventional cloning strategy, which contained 3.2 kb of 5′ DNA fragment upstream of the transcription initiation site, 172 bp of exon 1, the full length of intron 1 (1.4 kb), and 7 bp of exon 2 preceding the ATG starting codon of *Kera*, and an *IRES-NLS-Cre* minigene. The minigene was released by Not1 and Fsp1 restriction enzyme digestion and used in the microinjection of fertilized eggs by Transgenic Core at the Children’s Hospital of Cincinnati.

## Methods

### Preparation of *Kera-Cre* driver mice

Figure 1 showed the minigene construct used to create *Kera-Cre* (*KC*) driver mice. The minigene contains a 3.2 kb DNA fragment 5′ of the transcription initiation site of *Kera*, exon 1 (172 bp), intron 1 (1.4 kb), exon 2 (7 bp) preceding the ATG starting codon of *Ker*a mRNA, and a *Cr*e minigene with IRES (internal ribosome entry site) and NLS (nuclear localization sequences) followed by a SV40 polyadenylation signal [4]. The minigene was released from the plasmid by Not1 and Fsp1 restriction enzyme digestion and used in microinjection of fertilized FVB/N eggs. Twenty-three independent transgenic mouse lines (F0) were obtained and crossed with *Rosa26R* reporter mice [3]. Functional F1 offspring from each transgenic line was identified by X-gal staining. Six founders did not transmit the transgene to their offspring by polymerase chain reaction (PCR) genotype; six lines did not express the *Cr*e minigene and failed to recombine the *R26R* allele to express the *LacZ* reporter gene. These mouse lines were not examined further. Eleven transgenic founder lines harboring the *Kera-Cre* transgene were divided into three categories based on the population of βgal (β-galactosidase) positive cells in the cornea. Three mouse lines showed very low numbers of X-gal positive cells in the cornea. Five founder lines showed moderate positive cells, and three mouse lines showed very high numbers of positive cells in the cornea. To further verify the ocular tissues that express the transgene, the histological analysis of whole-mount X-gal stained eyes confirms that X-gal positive cells are found in the corneal stroma and not in the corneal epithelium and endothelium. One strong line (KC4.3) and one moderate line (KC4.1) were used in present studies. Both lines show the same promiscuous recombination of *LoxP* modified alleles during gametogenesis. Detailed cell lineage analyses using these *Cre* drivers are in progress and will be reported in the future (manuscript in preparation).

### Breeding of experimental mice

All animal protocols were approved by the Institutional Animal Care and Use Committee of the University of Cincinnati (Cincinnati, OH). The *Kera-Cre/ZEG*, *K12-Cre/ZEG*, *BF1-Cre/EGFP-R*, and *Wnt1-Cre/ZEG* mice were generated by crossing the female *ZEG* [5] and *EGFP-R* (a mouse line similar to *ZEG* except there is a *CAT* [chloramphenicol acetyl transferase] minigene in lieu of *LacZ* preceding the *EGFP*) [6] reporter mice with *Kera-Cre*, *K12-Cre*, *BF1-Cre* [7], and *Wnt1-Cre* [8], respectively. The female reporter mice were kept with males for continuous mating, and the number of pups was recorded. In some studies, *floxed-Smad4* (*Smad4^f/f^*) [9] and *floxed-Tbr2* (*Tbr2^f/f^*) mice [10,11] were used as reporter mice in lieu of *ZEG* and *EGFP-R* mice. For breeding the second generation, male double transgenic *Kera-Cre/ZEG*, *K12-Cre/ZEG*, *BF1-Cre/EGFP*, and *Wnt1-Cre/ZEG* mice were mated with wild type female *C57BL/6* mice. The expression of *EGFP* in pups from each transgenic line was examined under a ZEISS stereomicroscope (Carl Zeiss, Inc., Thornwood, NY) with epifluorescence attachment. The photos were taken under ultraviolet (UV) light with a GFP470 filter using an AxioCam (Carl Zeiss, Inc.). The opposite mating direction (female *Kera-Cre/ZEG*, *K12-Cre/ZEG*, and *Wnt1-Cre/ZEG* mated to a male *C57BL/6*) was also performed.

**Figure 2:**
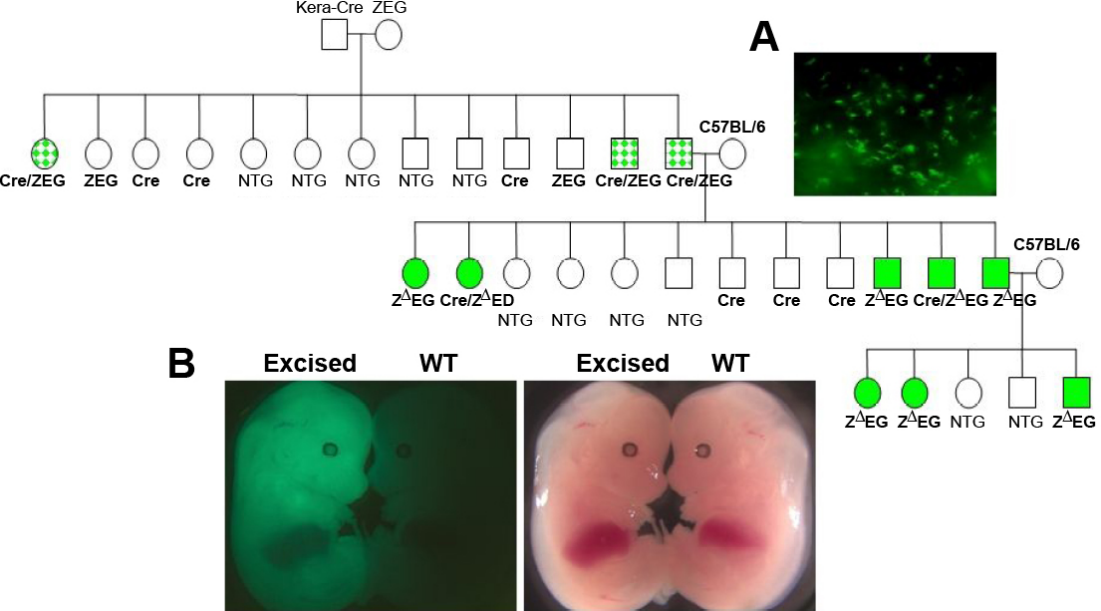
Pedigree analysis of the *Kera-Cre* transgenic mice mated with *ZEG* mice. *Kera-Cre* transgenic mice were crossbred with *ZEG* mice. Offspring from the mating male bitransgenic offspring (*Kera-Cre/ZEG*) with wild type *C57BL6* mice, which carried the *ZEG* transgene, were whole body green regardless of the presence or absence of the *Cre* transgene. Symbols: squares, male; circles, female; dotted square and circle, bitransgenic mice with cornea-specific *EGFP* expression; filled square and circle, *Z^∆^EG* mice with whole body *EGFP* expression. Non-transgenic animals were indicated with blank squares or circles. (**A**) Green fluorescence was observed in the cornea of a two-month-old *Kera-Cre/ZEG* mouse. (**B**) *EGFP* expression in E14.5 embryos from the timed mating of a male bitransgenic *Kera-Cre/ZEG* with a *C57BL/6* female was photographed using white light (right) and UV light (left) under a ZEISS fluorescent stereomicroscope. In each photo, the animal on the left is the *Z^∆^EG* embryo and the animal on the right is the wild type. Both photos showed identical animals (same embryo and same wild type animal) and just used different light sources to take each picture.

**Figure 3:**
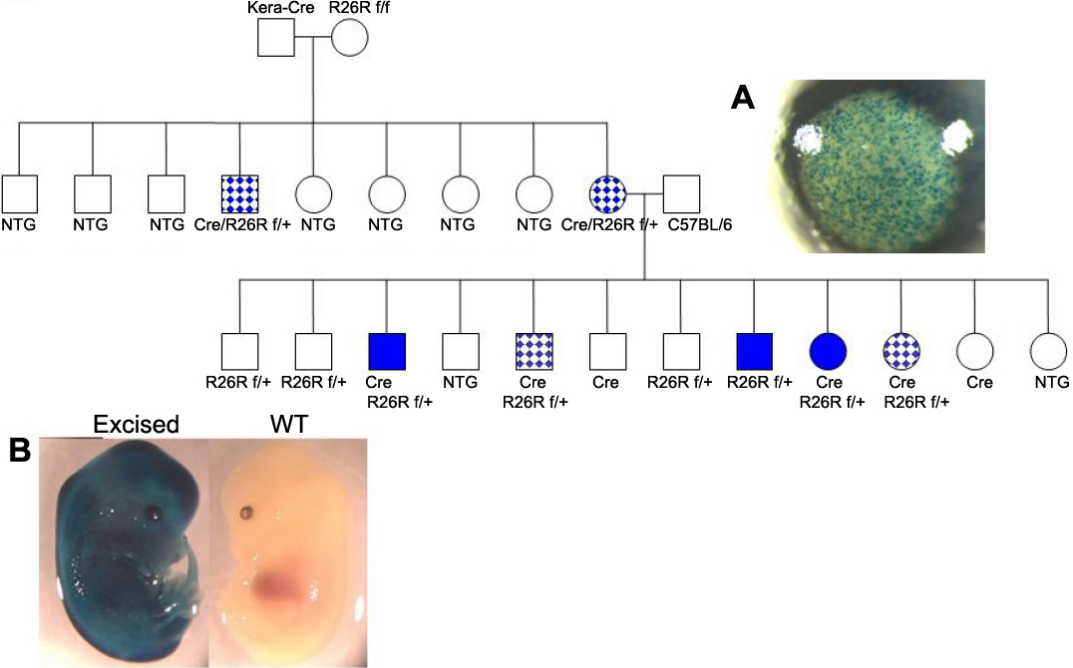
Pedigree analysis of the *Kera-Cre* transgenic mice mated with *Rosa26R* homozygous (*R26R*) mice. Squares stood for males, and circles stood for female animals; mosaic circles and squares indicate female and male animals, respectively, with a tissue specific manner of expressing *Cre* in the corneal stroma. The solid squares and circles indicate male and female animals, respectively, with expressing whole body blue by X-gal staining. Genotypes of experimental mice were determined by PCR of tail DNA as indicated under each of individual mice. *Cre* or *R26R ^f/w^* indicates single heterozygous transgenic mice harboring the *Cre* or *Rosa26R* gene alone; *Cre/Rsa26R^f/w^* indicates double transgenic mice harboring both *Cre* and heterozygous *Rosa26R* alleles and a wild type (*w*) *Rosa* allele. Non-transgenic animals were indicated with open squares or circles. (**A**) Detection of *Cre*-mediated recombination in the corneal stroma from double transgenic *Kera-Cre/Rosa26R^f/w^* mice is shown. Whole-mount X-gal staining was performed on the eye and hind limb from a two-month-old double transgenic *Kera-Cre/Rosa26R^f/w^* mouse. (**B**) X-gal staining in E14.5 wild type (WT) embryo and transgenic embryos with excision of the reporter gene is shown. Embryos were collected at E14.5 from *C57BL/6* female mated with male double transgenic *Kera-Cre/R26R* mouse. The β-gal activity was detected by whole-mount X-gal staining.

### Genotyping of transgenic mice and genetic analysis of recombination

Genotyping of mice was performed by PCR of tail DNA. For PCR detection of the *Kera-Cre* transgene, tail DNA was subjected to 35 amplification cycles with the following steps: 30 s at 94 °C; 30 s at 68 °C; 45 s at 72 °C, and a final amplification step of 5 min at 72 °C. The primers used were 5′-GTC GGT CCG GGC TGC CAC GAC C-3′ and 5′-GCA ATG GTG CGC CTG CTG GAA G-3′, which resulted in the amplification of a 722-bp *Cre* fragment. For PCR detection of the *K12-Cre* transgene, tail DNA was subjected to 35 amplification cycles with the following steps: 30 s at 94 °C; 30 s at 65 °C; 1 min at 72 °C, and a final amplification step of 5 min at 72 °C. The primers used were 5′-AAA GCG CAT GCT CCA GAC TGC C-3′ and 5′-GCA ACA GAG TTA GGA CTT GAA CCC-3′, which resulted in the amplification of a 1300-bp PCR product. Five different reporter lines were used in this study. The first reporter line, *Rosa26R* [3], *LacZ* driven by a *Rosa26* promoter, is expressed following a *Cre*-mediated recombination. The second and third reporter mouse lines (*ZEG* and *EGFP-R*) use a strong CMV (cytomegarovirus) enhancer and the α-actin promoter to express *LacZ* of *ZEG* and (*CAT*) of *EGFP-R* minigenes before Cre-mediated recombination and *EGFP* (enhance green fluorescence protein) afterward [5,6]. *Floxed-Smad4* and *floxed-Tbr2* mice served as reporter mice to detect recombination caused by the promiscuous expression of *Kera-Cre* transgene using PCR of tail DNA. The primers used were 5′-GGG CAG CGT AGC ATA TAA GA-3′ (primer b) and 5′-GAC CCA AAC GTC ACC TTC AC-3′ (primer c), which resulted in the amplification of a 450 bp and 390 bp PCR product for the *LoxP* modified and the wild type allele of the *Smad4^f/w^* mouse, respectively. The 5′-AAG AGC CAC AGG GTC AAG CA-3′ (primer a) and 5′-GAC CCA AAC GTC ACC TTC AC-3′ (primer c) primers were used to detect excised *Smad4^f^* allele for a 500-bp PCR product resulted from the amplification of the recombined *floxed-Smad4* allele.

**Table 1:** Excision of *floxed* alleles during gametogenesis of double transgenic *Cre/floxed* mice.

**Father**	**Mother**	**Number of Cre^tg^/floxed mice**		**Number of Cre^0^/floxed mice**		**Percent penetrance##**
		**Total**	**Δ**	**Total**	**Δ**	
*KC4.3/ZEG*	*C57BL/6*	10	10	10	10	100
*KC4.1/ROSAR*	*C57BL/6*	4	4	6	6	100
*KC1/ZEG*	*C57BL/6*	15	15	16	16	100
*C57BL/6*	*KC4.3/ROSAR*	4	2	1	1	60
*K12^Cre/w^/ZEG*	*C57BL/6*	NA*	NA1	13	6	46
*C57BL/6*	*K12^Cre/w^/ZEG*	NA*	NA1	27	2	7
*BF1^Cre^/R26R*	*CD-1*	7	5	9	6	69
*C57BL/6*	*Wnt1-Cre/R26R*	1	0	5	0	0
*Wnt1Cre/R26R*	*C57BL/6*	1	0	7	0	0
*Tbr2^f/f^*	*KC4/Tbr2^f/w^*	18#	5	26#	5	23
*Smad4^f/f^*	*KC4.3/Smad4^f/w^*	6#	2	7#	3	38

Double transgenic mice were cross bred with wild type mice, and homozygous *Tbr2^f/f^* and *Smad4^f/f^* mice. The excision event was determined by the expression of respective reporter gene activities or by PCR of the excised *floxed* alleles as described in Methods. Cre^Tg^: hemizygous *Cre* transgenic mice; Cre^0^: non-*Cre* transgenic mice. The asterisk indicates that the ZEG transgene is on chromosome 11, the same as *Krt12*. Thus, the ZEG allele is always co-segregated with the *Krt12^Cre^* allele. The sharp (hash mark) indicates homozygous floxed *Tbr2* and *Smad4* alleles and the double sharp indicates that the percent of penetrance is calculated by number of mice with excised floxed allele divided by the total number of mice carrying floxed allele in each experimental group.

### Reverse transcription polymerase chain reaction for the detection of keratocan, keratin 12, and *Cre* mRNAs in testis

Total RNAs were isolated from four pooled corneas and individual testis of two adult *Kera-Cre* mice (three months old) with TRIzol Reagent (Invitrogen, Carlsbad, CA), dissolved in DEPC-water, and stored at −80 °C until use. Ten micrograms (testis) and 3.5 μg (cornea) total RNA were annealed to oligo-dT and reverse transcribed with avian reverse transcriptase kits from Promega (Madison, WI) according to the manufacturer’s instruction. The single strand cDNAs were subjected to PCR with primer pairs for keratocan: forward (F) 5′-GAT TAC CAG CCA ACA CCA TGC AAC TC (exon 2), reverse (R) 5′-CAT CCA GAC GCA GGT AGC TCA GTT GT (exon 3); *Kera-Cre*: F 5′- TAA CAC CAG CCA CAG GAC TCA AC (exon 1 of *Kera*), R 5′- ATG GGG TAC CTT CTG GGC ATC CTT (*IRES*); K12 (exon 1–3): F 5′ CTG GCA ATG ATG GAG GTC TTC T (exon 1), R 5′ GCT TCC AGG TCG GCT CTA GTC A (exon 3); *K12*: F 5′- AAC CGC AGA CAC CAT CAG TCG ATT (exon 7), R 5′- TAA AGA AAT GGT CCA GGC GAC CAG (exon 8); *GAPDH*: F 5′ AAG GTG GTG AAG CAG GCA TCT GAG (exon 6), R 5′-TCT TAC TCC TTG GAG GCC ATG TAG (exon 7).

PCR was performed at 95 °C for 2 min, 40–45 cycles at 94 °C 25 s, 56/58 °C 25 s, 72 °C 1 min/Kb, then 72 °C for 10 min.

### Preparation of testicular cells

The testes from a *Kera-Cre/Smad4 ^f/w^* bitransgenic mouse were dissected in a Petri dish containing an ice-cold separation medium (4 mM L-glutamine, 1.5 mM sodium pyruvate, 10% fetal calf serum, and 75 µg/ml ampicillin in DMEM) [12-14]. The testis was decapsulated and was forced into a 15-ml Falcon tube containing 5 ml of the ice-cold separation medium. Then, 0.25 ml of collagenase (2 mg/ml) was added to the tube with the decapsulated testes, and incubation was performed for 5 min at 37 °C with shaking. The seminiferous cords were washed twice in 10 ml of the separation medium, resuspended in 12 ml of a separation medium containing 2.5 µg/ml trypsin and 1 U/ml DNase I, incubated for 10 min at 37 °C, and transferred onto ice. A Pasteur pipette was used to disintegrate seminiferous cords into single cells and then filtered through a 40 µm nylon mesh. Cell suspension was washed twice with the separation medium (centrifugation at 200–300 xg) and counted.

### Enrichment of spermatogenic cells

To increase the proportion of secondary spermatocytes before FACS analysis, the cell suspension isolated from testis was centrifuged on a discontinuous Percoll gradient prepared as described in the following. The 90% of isoosmotic Percoll was used to prepare 50%, 40%, 36%, 33%, 30%, and 20% Percoll solution by dilution with PBS. After centrifugation (800 xg, 30 min), fractions of 33% and 36% Percoll density were collected and washed twice with DMEM [15].

### May–Grünwald-Giemsa staining

The May–Grünwald-Giemsa staining technique [16] was used to identify various kinds of germ cells (spermatogonia, primary spermatocytes, and spermatids)

### Flow cytometry analysis and sorting

For FACS analysis, testicular cells were brought to a concentration of 4×10^6^ cells/ml in the separation medium and diluted 1:1 with propidium iodide solution (10 mM Tris, pH 8, 1 mM NaCl, 0.1% Nonidet P-40, 0.7 mg/ml RNase A, and 0.05 mg/ml propidium iodide). Cells were analyzed by a Becton, Dickinson FACS Diva (San Jose, CA) within 2 h from staining. Excitation was at 488 nm using an argon ion laser, and emission was collected at 585±21 nm. For recombination analysis, cells from designated populations (1N, 2N, and 4N) were sorted into a tube containing 1 ml PBS and collected onto a 0.2 μm filter. DNA was extracted from the cells and subjected to PCR. DNA histograms were analyzed using FloJo (TreeStar, Ashland, OR).

### X-gal staining

*Kera-Cre/Rosa26R* double transgenic and *Rosa26R* single transgenic control mice were euthanized and perfused with 4% paraformaldehyde in PBS, and testes were removed. Testes were fixed again for 2 h at room temperature in 4% paraformaldehyde and then incubated overnight at 37 °C in a solution of 5-bromo-4-chloro-3-indolyl-β-galactopyronoside (X-gal) at a final concentration of 1 mg/ml made from a 100 mg/ml stock in dimethylformamide with 4 mM K_3_Fe(CN)_6_, 4 mM K_4_Fe(CN)_6_, 2 mM MgCl_2,_ 0.2% NP-40, and 0.2% sodium deoxycholate in PBS. After staining, testes were rinsed with PBS and then embedded in paraffin. Sections (5 μm) were deparaffinized, counterstained with neutral red (5%), and examined with a Nikon E800 microscope (Nikon, Melville, NY).

### Results and Discussion

*Keratocan-Cre* (*Kera-Cre*) transgenic driver mice intended for corneal stromal keratocyte-specific gene ablation were bred with reporter mice to prepare double transgenic *Kera-Cre/ZEG* and *Kera-Cre/Rosa26R* mice that showed corneal stroma-specific expression patterns of *EGFP* (Figure 2A) and *LacZ* (Figure 3A), respectively. Sibling breeding of double transgenic mice of *Kera-Cre/ZEG* or *Kera-Cre/Rosa26R* was employed to increase the colony size of such double transgenic mice. Surprisingly, animals (Figure 2B) derived from *Kera-Cre/ZEG* mice expressed whole body green fluorescence while those derived from *Kera-Cre/Rosa26R* mice were whole body blue by X-gal staining (Figure 3B). A similar phenomenon was observed when such double transgenic mice were cross-bred with wild type *C57BL/6* mice. Pedigree analysis revealed that male double transgenic *Kera-Cre/ZEG* and *Kera-Cre/Rosa26R* mice bred with female *C57BL/6* mice produced offspring containing *ZEG* and *Rosa26R* alleles, which showed 100% penetrance of whole body green fluorescence and blue by X-gal staining, respectively, even when the *Cre* transgene was absent in the progeny (Table 1). However, in the opposite mating direction (female *Ker-Cre/Rosa26R* mated to a male *C57BL/6*), the offspring had a lower incident of whole body blue (Figure 3 and Table 1). The observations suggest that Cre activity may take place during gametogenesis. To explore commonality of such promiscuous Cre activity, other tissue-specific *Cre* driver mouse lines (*Keratin12-Cre*, *K12-Cre*; *Foxg1-Cr, BF1-Cre*; and *Wnt1-Cre*) were analyzed (Table 1). A similar pattern was observed in the *K12-Cre* and *BF1-Cre* mouse line albeit at a lower frequency but not in the *Wnt1-Cre* mouse line, indicating that promiscuous *Cre* activity is common in different tissue-specific *Cre* driver mouse lines. To elucidate whether different *LoxP* modified alleles may also be susceptible to promiscuous Cre activities, *Kera-Cre* driver mice were mated with *Smad4 ^f/f^* and *Tbr2 ^f/f^* mice to generate double transgenic mice of *Kera-Cre/Smad4 ^f/w^* and *Kera-Cre/Tbr2 ^f/w^* mice, respectively. PCR genotyping of tail DNA of the tails of offspring revealed that the excision of one floxed alleles had occurred in the progeny of double transgenic mice (data not shown). These results indicated that promiscuous Cre activity of the transgenic *Kera-Cre* mouse line took place regardless of the position of the floxed allele in the genome (Table 1).

**Figure 4:**
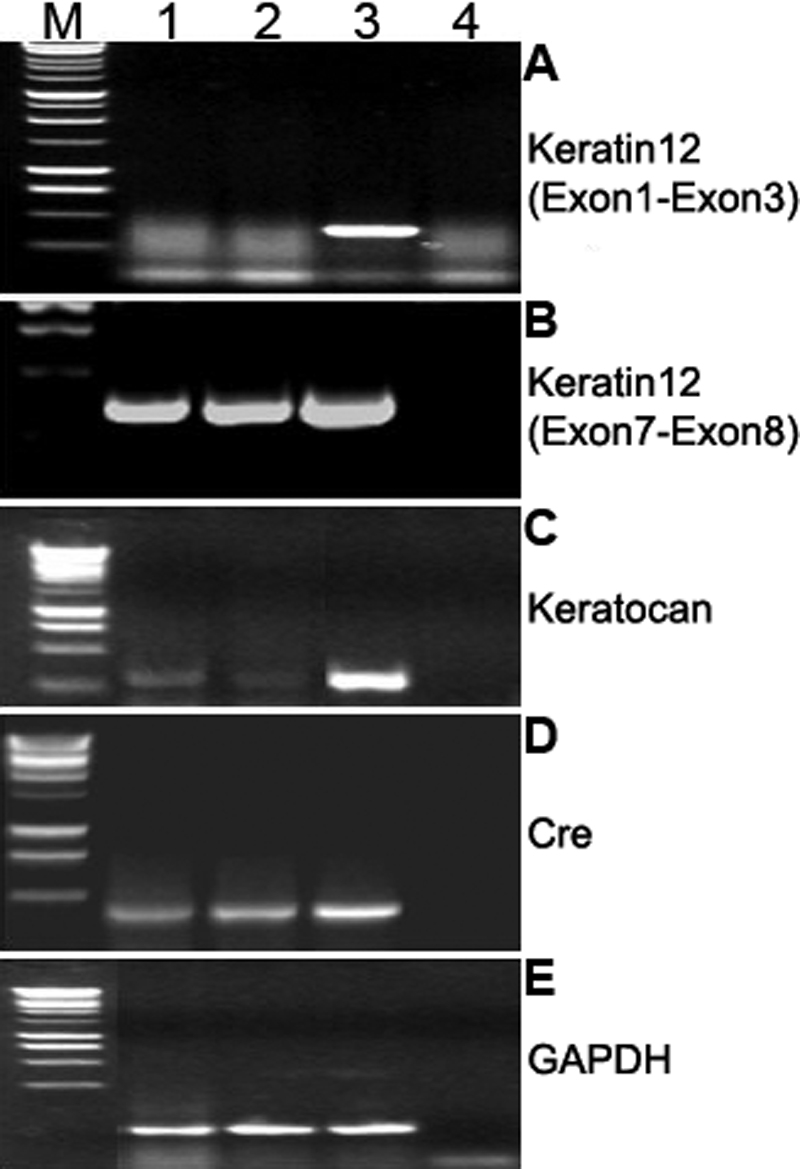
Reverse transcription polymerase chain reaction for detection of keratocan, keratin 12 and Cre mRNAs in testis. Total RNAs were isolated from four pooled corneas and individual testis of two *Kera-Cre* mice with TRIzol Reagent (Invitrogen), and then the total RNAs were dissolved in DEPC-Water and stored at −80 °C until use. Ten micrograms (testis) and 3.5 μg of total RNA were annealed to oligo-dT and reverse transcribed with kits from Promega according to the manufacturer’s instruction. Single stranded cDNA was subjected to PCR reactions using primer pairs for *keratocan* (*Kera*), *keratin 12* (*K12*), *Cre recombinase* (*Cre*), and *glyceraldehydes phosphate dehydrogenase* (*GAPDH*) as described in Methods. **A**: Use of primers in exons 1 and 3 of *Krt12* generates a 408 bp RT–PCR product with total RNA from the cornea, but not that from testis; **B**: use of primers in exons 7 and 8 of *Krt12*, a 410 bp RT–PCR product was detected with RNA from both corneas and testis; **C**: use of primers in exons 2 and 3 of *Kera* produces a 350 bp RT–PCR product with RNA from cornea and testis; **D**, Use primer of exon 1 of *Kera* and primer of IRES yields a 480 bp RT–PCR product in RNA from both cornea and testis of *Kera-Cre* mice; **E**: primers of exon 6 and 7 of *GAPDH* gene, a 200 bp transcript was used as a positive control RT–PCR with RNA from both cornea and testis. Lane 1, testis 1; lane 2, testis 2; lane 3, cornea; lane 4, control (no cDNA added); M, 1 kb DNA markers.

Reverse transcription polymerase chain reaction (RT–PCR) with specific primers for transcripts of cornea-specific keratin 12 and keratocan was performed to verify whether the observed promiscuous Cre activities were derived from transcription activities of such authentic cornea-specific genes during gametogenesis from the leaky transcription of *Cre*-minigenes driven by the tissues-specific promoters. Figure 4 showed that both *keratin 12* (*K12*; exon 7 and 8 primers) and keratocan (primers of exon 2 and 3) transcripts were detected in total RNA prepared from the testis and corneas of adult three-month-old mice. It is of interest to note that the use of primers in exon 1 and 3 of the *K12* gene detected K12 mRNA in the cornea but not in testis. The presence of *Cre* transcripts were also detected in the cornea and testis of double transgenic K*era-Cre/ZEG* mice. To verify whether *K12* and keratocan proteins might be synthesized by testicular cells, immunohistochemistry failed to detect the presence of keratocan and K12 in testis with antibodies against keratocan and keratin 12, respectively (data not shown). The observations suggested that tissue-specific genes may be transcribed without the production of significant amounts of the respective protein during gametogenesis. We have previously demonstrated that alternatively spliced keratin 12 transcripts from a single promoter are detected in numerous tissues demonstrated by northern hybridization, but only the corneal epithelium has the correctly spliced transcript with an open reading frame for the production of functional keratin 12 protein [17]. The knock-in strategy was used to prepare the *K12-Cre* mice in which an *IRES-Cre* minigene was inserted immediately following the stop codon in exon 8 of the *K12* gene. Thus, the presence of IRES in the modified *K12* transcript allows the synthesis of *Cre* recombinase while no functional keratin 12 was produced. It is worthy to caution that *Cre* expression should be checked in the use of the knock-in strategy using the *IRES-Cre* minigene targeted for tissue-specific gene ablation of the *Cre/LoxP* strategy. Furthermore, tissue-specific promoter Cre minigenes may be transcribed if the authentic tissue-specific genes may be promiscuously and transiently transcribed during gametogenesis, a situation which then leads to the synthesis of Cre recombinase and the excision of *LoxP* modified alleles.

**Figure 5:**
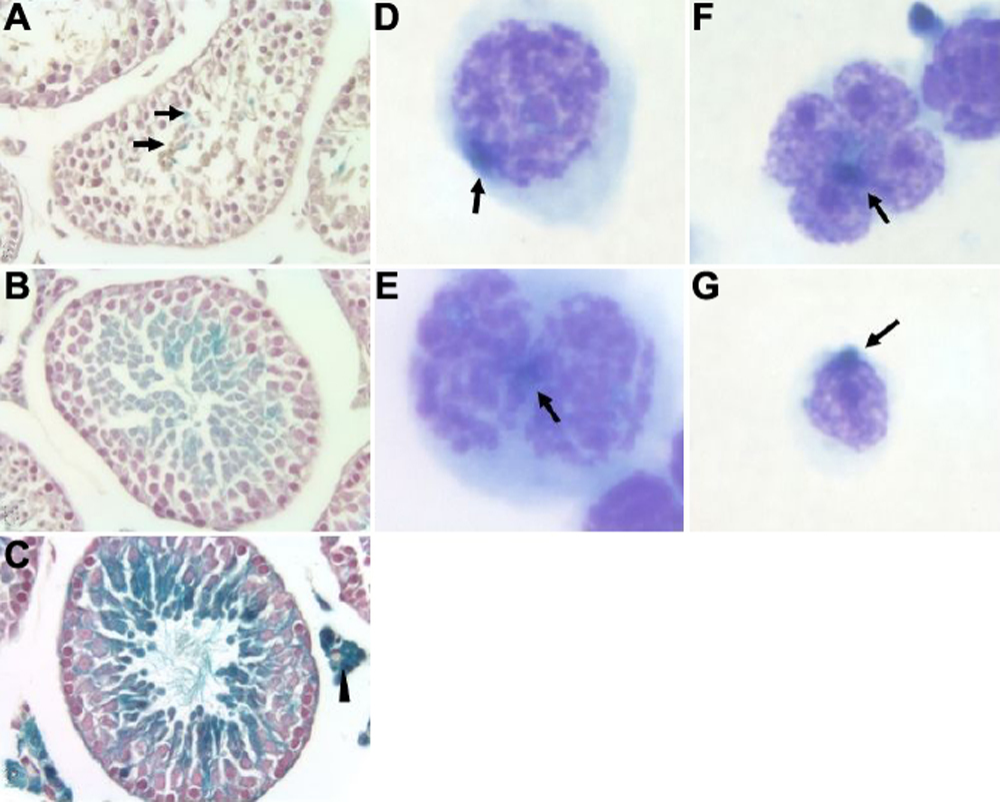
Expression of the *LacZ* gene in double transgenic *Kera-Cre/Rosa26R* mice during spermatogenesis. Whole mount X-gal staining of testes collected from 20-(**A**), 30-(**B**), and 60 day-old (**C**) bitransgenic mice showed positive reaction in primary and secondary spermatocytes and spermatids. No β-gal activity was found in adult single *Rosa26R* transgenic testes and 10-day-old testes of double *Kera-Cre/Rosa26R* mice (data not shown). Leydig cells (arrowheads) have endogenous β-gal activity. Testicular cell suspensions prepared from a *Kera-Cre/Rosa26R* mouse were subjected to May–Grünwald-Giemsa stain after X-gal staining. Positive X-gal staining (arrow) was observed in the primary spermatocytes (**D**), primary spermatocytes in meiotic division (**E**), spermatids (**F**), and round spermatids (**G**).

The whole body floxed allele recombination in the progeny from presumed tissue-specific *Cre* driver mice can be explained by the *Cre* expression in gametes, which causes the floxed allele to be recombined at this early stage of development as mentioned above. To further investigate this possibility and to determine when such excision events take place during spermatogenesis, *LacZ* expression was determined in the testes of *Kera-Cre/Rosa26R* mice at different ages by whole mount X-gal staining. No X-gal signal was found in testicular cells of single transgenic and 10-day-old double transgenic mice. X-gal positive cells were detected in the testes of 20- (Figure 5A), 30- (Figure 5B), and 60-day-old mice (Figure 5C). In the adult testis, β-galactosidase activity was detected in primary and secondary spermatocytes and spermatids but not in spermatogonia, indicating that the Cre activity is present at meiosis I where primary spermatocytes form from spermatogonia. The evaluation with May–Grünwald-Giemsa staining of the testicular cells showed that X-gal positive cells were found in primary spermatocytes and spermatids of the *Kera-Cre/Rosa26R* mouse (Figure 5D,E,F,G).

**Figure 6:**
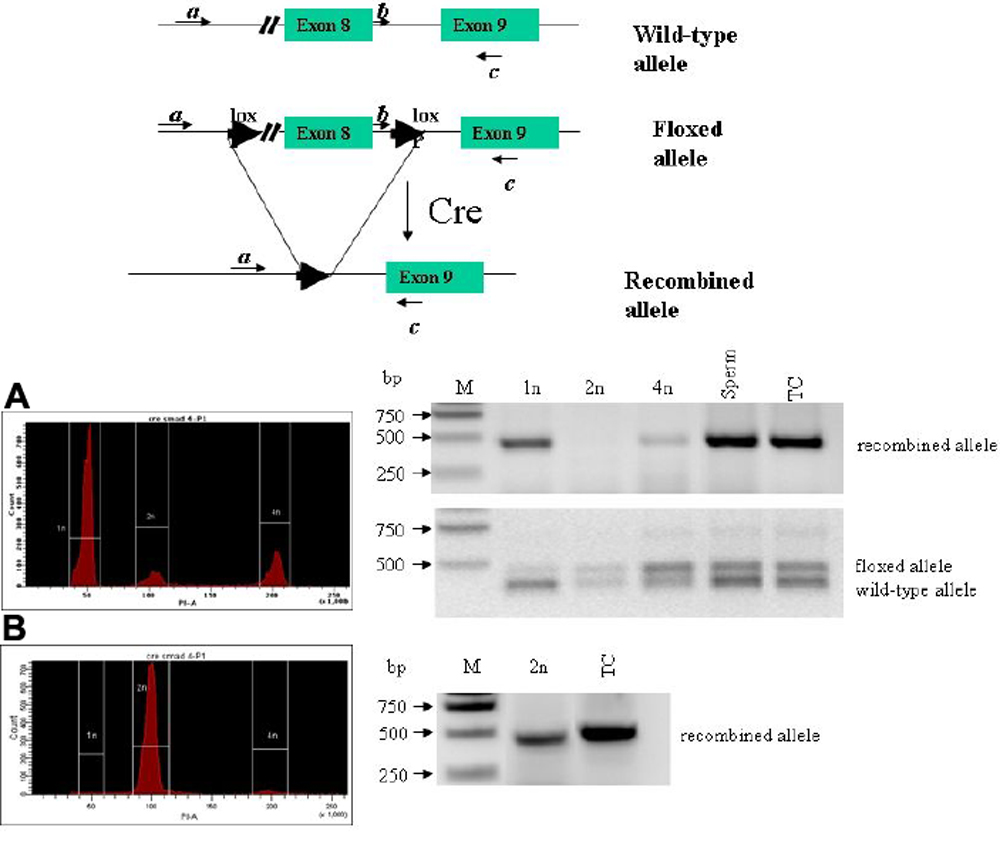
*Cre*-mediated recombination of the *Smad4^f^* allele in spermatogenic cells of double transgenic *Kera-Cre/Smad4^f/wt^* mouse. (**A**) Histograms of flow cytometry of testicular cell suspension obtained from a 60-day-old double transgenic *Kera-Cre/Smad4^f/w^* mouse showed a distribution of 65%, 9%, and 17% for 1N, 2N, and 4N cells, respectively. PCR of genomic DNA from 1N and 4N cells showed excision of the *Smad4^f^* allele but not from 2N cells. (**B**) Histogram showed enrichment of 2N cells by Percoll gradients as described in Methods. PCR analysis of these enriched 2N cells demonstrated excision of the *Smad4^f^* allele. Primers b and c identify wild type *Smad4* (390 bp) and floxed *Smad4^f^* allele (450 bp) whereas primers a and c identify the excised *Smad4^f^* allele (500 bp). TC, testicular cells; M, DNA marker.

**Figure 7:**
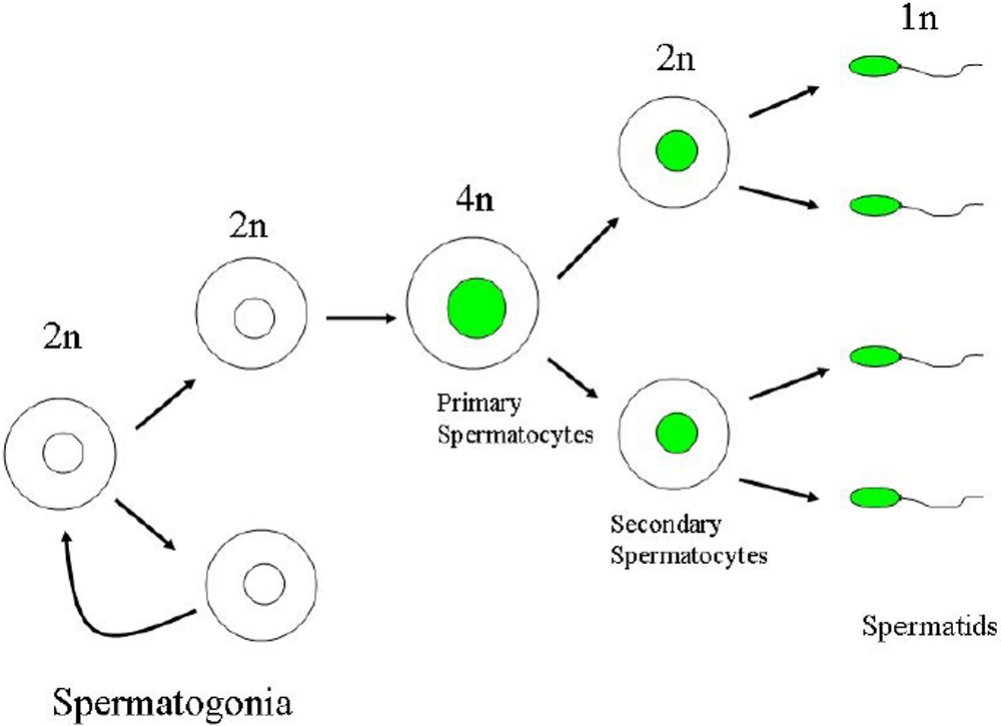
Hypothetical cryptic Cre activity during gametogenesis. It is hypothesized that the expression of *Cre* occurs when progenitor germ cells commit terminal differentiation and enter gametogenesis at which time floxed allele(s) may undergo *Cre*-mediated recombination.

The recombination of the floxed allele by Cre recombinase was detected in the sperm and testicular cells of double transgenic *Kera-Cre/Smad4 ^f/w^* mouse (Figure 6), indicating that Cre activity was activated during gametogenesis. To elucidate further, testicular cells from double transgenic *Kera-Cre/Smad4^f/w^* mice were subjected to cell sorting by flow cytometry following propidium iodide staining to separate cells by DNA content. Genomic DNA from haploid (1N, one copy of the genome), diploid (2N), and tetraploid cells (4N) were subjected to PCR analysis. In the adult mouse (two months old), the testis exhibited 1N, 2N, and 4N subpopulation around 65%, 9%, and 17%, respectively. Recombination of *Smad4^f^* allele was found in 1N and 4N cells but not in the 2N cells (Figure 6A). The 2N cells in the adult testis consist of testicular somatic cells (e.g., Sertoli, Leydig, and peritubular cells) and a small number of spermatogonia and secondary spermatocytes. Since meiosis I is immediately followed by meiosis II, the number of secondary spermatocytes (2N) in testicular cell suspension is too little to detect recombined *Smad4^f^* allele. Percoll discontinuous gradient centrifugation was used to enrich 2N testicular cells. Cytometry analysis indicated a reduction of 1N cells and an increase of 2N cells. The PCR results of these 2N cells indicated that recombination of *Smad4^f^* allele occurred (Figure 6B). These results were consistent with the notion that Cre activity existed when spermatogonia committed terminal differentiation of meiosis (Figure 7).

A previous report demonstrated that *Cre* expression occurred in late-stage oocytes of the *Keratin K5Cre* transgenic line leading to the constitutive recombination of floxed alleles in offspring [18]. The use of northern hybridization recently demonstrated that *Foxg1* (*BF1*) was expressed in the testis and served as a corepressor of the androgen receptor. However, such a *Foxg1* transcript was not detected in the rodent testis [19]. In our previous studies of keratocan and K12 keratin, northern hybridization and immunohistochemistry with peptide antibodies were used to determine the stromal keratocyte-specific and corneal-epithelium specific expression patterns of both genes, respectively [4,17,20]. *Kera-LacZ* transgenic mice also showed corneal keratocyte-specific expression of reporter β-galactosidase activities in adults [21]. It is worthy noting that the ablation of keratocan and K12 keratin did not have adverse effects in the fertility of either sex of the homozygous null mice [22,23]. Keratocan is an extracellular matrix component of the small leucine-rich proteoglycan family while K12 keratin is a component of the corneal epithelium intermediate filament. Both proteins do not have any known functions in gametogenesis. The results support the notion that both genes are cornea-specific for the synthesis of keratocan and keratin 12 in adult mice. The promiscuous expression of keratocan, keratin 12, and transgenes driven by the respective promoters may result from noise in genetic networks as suggested by Paulsson [24]. It is of interest to note that these two genes encode for structural proteins thus they may not be subjected to strict regulation as that of genes encoding regulatory proteins such as Wnt. Thus, these two genes’ uncontrolled expression may have serious impacts on the organism during development [25]. Therefore, it is advisable that the most sensitive methods such as RT–PCR is used to detect the existence of minute amount of transcripts in testis before the generation of Cre driver mice with such promoters of so-called tissue-specific genes outside the gonads in adults. Alternatively, tamoxifen inducible CrePR/LoxP [26] and doxycycline inducible tissue-specific promoter-*rtTA/tetOCre/LoxP* systems may be used to avoid such promiscuous Cre activities in the simple *Cre/LoxP* system described in the present studies. It is of interest to note that in our preliminary studies using triple *K12-rtTA/tetO-Cre/X^f/f^* (floxed-X gene of interest), the cryptic Cre activity during gametogenesis can be avoided and the ablation of *LoxP* modified allele only takes place upon doxycycline induction (data not shown).

Our study demonstrated that both genders of the first generation of bitransgenic mice (e.g., *Kera-Cre/ZEG, K12-Cre/ZEG, or BF1-Cre/ZEG* from breeding of respective single transgenic mice) showed tissue-specific patterns of *EGFP* while their progenies might show whole body green by cryptic *Cre*-mediated recombination, and there is a different rate of germ cell recombination when different breeding strategy is selected. The reason for the higher penetrance of promiscuous *Cre* recombination observed in male double transgenic mice than in the female double transgenic mice can be in part explained by the presence of a cytoplasmic bridge formed among spermatogenic cells through which Cre recombinase can diffuse from one haploid cell to another during spermatogenesis whereas such a cytoplasmic bridge does not exist during oogenesis [27]. The present study is the first to conclusively demonstrate promiscuous Cre activity during gametogenesis and to provide useful information to alert other investigators using the *Cre/LoxP* system in conditional gene ablation.

It should be cautioned that PCR genotyping of excision event must be performed to assure that homozygous *Cre/X^f/f^* mice are obtained instead of hemizygous *Cre/X^f/Δ^* mice, when sibling breeding of *Cre/X^f^* bitransgenic mice is used. It is very likely that the so-called tissue-specific genes in adult mice may be activated and lead to undesirable promiscuous Cre activities during gametogenesis. Our studies are of paramount importance in avoiding the misinterpretation of phenotypes resulting from conditional gene ablation with *Cre-LoxP* strategies. For example, when a double transgenic *Kera-Cre/Smad4 ^f/w^* male is mated with a *Smad4 ^f/f^* female, one may assume to obtain about 25% of progenies as *Kera-Cre/Smad4 ^f/f^* mice, however, one allele of *Smad4^f^* may have been deleted systemically among the 25% of the offspring that are actually hemizygous *Smad4^f/Δ^* mice albeit both alleles are deleted in cornea stromal keratocytes. It is not uncommon that a haploid deficiency exists in the heterozygous gene knockout mice [28,29]. Therefore, to avoid the unexpected deletion of the gene of interest in the *Cre/LoxP* system, it is suggested that researchers should be aware of this pitfall when *Cre/LoxP* conditional gene ablations are prepared by using female double transgenic *Cre/floxed* heterozygous mice to mate with male *floxed* homozygous mice so that more experimental mice of true tissue-specific gene ablation can be obtained.
